# Impaired ROS Scavenging System in Human Induced Pluripotent Stem Cells Generated from Patients with MERRF Syndrome

**DOI:** 10.1038/srep23661

**Published:** 2016-03-30

**Authors:** Shih-Jie Chou, Wei-Lien Tseng, Chien-Tsun Chen, Yu-Fen Lai, Chian-Shiu Chien, Yuh-Lih Chang, Hsin-Chen Lee, Yau-Huei Wei, Shih-Hwa Chiou

**Affiliations:** 1 Institute of Pharmacology, Taipei, Taiwan; 2 Institute of Clinical Medicine, Taipei, Taiwan; 3 School of Medicine, National Yang-Ming University, Taipei, Taiwan; 4 Department of Medicine, Mackay Medical College, New Taipei, Taiwan; 5 Department of Medical Research, Taipei Veterans General Hospital, Taipei, Taiwan

## Abstract

Myoclonus epilepsy associated with ragged-red fibers (MERRF) is a mitochondrial disorder characterized by myoclonus epilepsy, generalized seizures, ataxia and myopathy. MERRF syndrome is primarily due to an A to G mutation at mtDNA 8344 that disrupts the mitochondrial gene for tRNA(Lys). However, the detailed mechanism by which this tRNA(Lys) mutation causes mitochondrial dysfunction in cardiomyocytes or neurons remains unclear. In this study, we generated human induced pluripotent stem cells (hiPSCs) that carry the A8344G genetic mutation from patients with MERRF syndrome. Compared with mutation-free isogenic hiPSCs, MERRF-specific hiPSCs (MERRF-hiPSCs) exhibited reduced oxygen consumption, elevated reactive oxygen species (ROS) production, reduced growth, and fragmented mitochondrial morphology. We sought to investigate the induction ability and mitochondrial function of cardiomyocyte-like cells differentiated from MERRF-hiPSCs. Our data demonstrate that that cardiomyocyte-like cells (MERRF-CMs) or neural progenitor cells (MERRF-NPCs) differentiated from MERRF-iPSCs also exhibited increased ROS levels and altered antioxidant gene expression. Furthermore, MERRF-CMs or -NPCs contained fragmented mitochondria, as evidenced by MitoTracker Red staining and transmission electron microscopy. Taken together, these findings showed that MERRF-hiPSCs and MERRF-CM or –NPC harboring the A8344G genetic mutation displayed contained mitochondria with an abnormal ultrastructure, produced increased ROS levels, and expressed upregulated antioxidant genes.

Human mitochondrial DNA (mtDNA) is consisted of 16,569 bp encoded 37 genes, which includes 13 polypeptides of the mitochondrial respiratory chain responsible for mitochondrial oxidative phosphorylation (OXPHOS) as well as 2 rRNAs and 22 tRNAs for mitochondrial protein synthesis^1^. Rearrangements of mtDNA, including deletions or mutations, result in a wide spectrum of clinical manifestations ranging from mild muscle weakness to fatal infantile diseases such as mitochondrial encephalomyopathy, lactic acidosis, stroke-like episodes (MELAS)^2^, and myoclonus epilepsy with ragged-red fiber (MERRF) disease^3^,^4^.

MERRF disease is a maternally inherited mitochondrial encephalomyopathy characterized by myoclonus epilepsy, generalized seizures, ataxia and myopathy^4^. Apart from the well-characterized syndrome in neural system, there are around 53% MERRF patient also suffered cardiomyopathy^5^. In over 80% of the cases, an A to G mutation at mtDNA 8344 disrupts the mitochondrial gene for tRNA-Lys, which is associated with severe defects in protein synthesis, leading to impaired OXPHOS^4^,^6^. mtDNA mutation-elicited oxidative stress, oxidative damage and altered gene expression are involved in the pathogenesis and progression of MERRF syndrome^7^,^8^. In previous studies, the autophagic pathway was stimulated in human cells harboring A8344G mutation, resulting in the degradation of heat shock protein 27 (Hsp27)^9^. In addition, excess reactive oxygen species (ROS) may damage voltage-dependent anion channels (VDAC), prohibitin, Lon protease, and aconitase in MERRF cells^8^. Moreover, high levels of matrix metalloproteinase 1 expression and activity may contribute to the cytoskeleton remodeling that is involved in the muscle weakness and atrophy of MERRF patients^8^. However, the detailed molecular mechanisms by which A8344G point mutation-induced ROS stress affects mitochondrial dynamics and progressive cell damage in MERRF syndrome remains unknown.

Recent progress in induced pluripotent stem cells (iPSCs) technology has facilitated the successful generation of human iPSCs via the forced expression of transcription factors such as OCT4, SOX2, KLF4, and c-MYC or OCT4, SOX2, LIN28 and NANOG in somatic cells. These cells have provided new opportunities for regenerative medicine and *in vitro* disease modeling^10^,^11^,^12^,^13^. Several attempts have been made to establish disease-specific human iPSCs (hiPSCs) from individual patients such as cells specific for neurodegenerative diseases, including amyotrophic lateral sclerosis (ALS), Parkinson’s disease (PD), Huntington’s disease (HD) and Alzheimer’s disease (AD)^14^,^15^,^16^,^17^. Moreover, patient-specific iPSC models have been established to study the mechanisms of mtDNA mutation-associated diseases such as MELAS syndrome and Pearson marrow pancreas syndrome (PS)^18^,^19^. The generation of patient-specific hiPSCs is undoubtedly an important tool to generate the pathogenic models of genetically inherited diseases.

In the present study, we aimed to establish a platform that serves as a disease model to elucidate the pathological role of mitochondrial ROS. We also explored the association of these stresses with mitochondrial dynamics. This study demonstrates that MERRF-hiPSCs carrying the mtDNA A8344G mutation express ES-like markers and can differentiate in to three germ layers. MERRF-hiPSCs exhibit impaired mitochondrial function and reduced growth. Furthermore, MERRF-derived cardiomyocytes contain fragmented and functionally impaired mitochondria. Moreover, we demonstrate that elevated ROS levels may be attributed to the changes in antioxidant gene expression in MERRF-hiPSCs, MERRF-hiPSC-derived cardiomyocytes and MERRF-hiPSC-derived neural progenitors. Overall, we generated MERRF-specific hiPSC as an *in vitro* models for investigating the pathophysiologic mechanism of mitochondrial diseases.

## Results

### Generation of hiPSCs from dermal fibroblasts of MERRF patients

**Figure 1:**
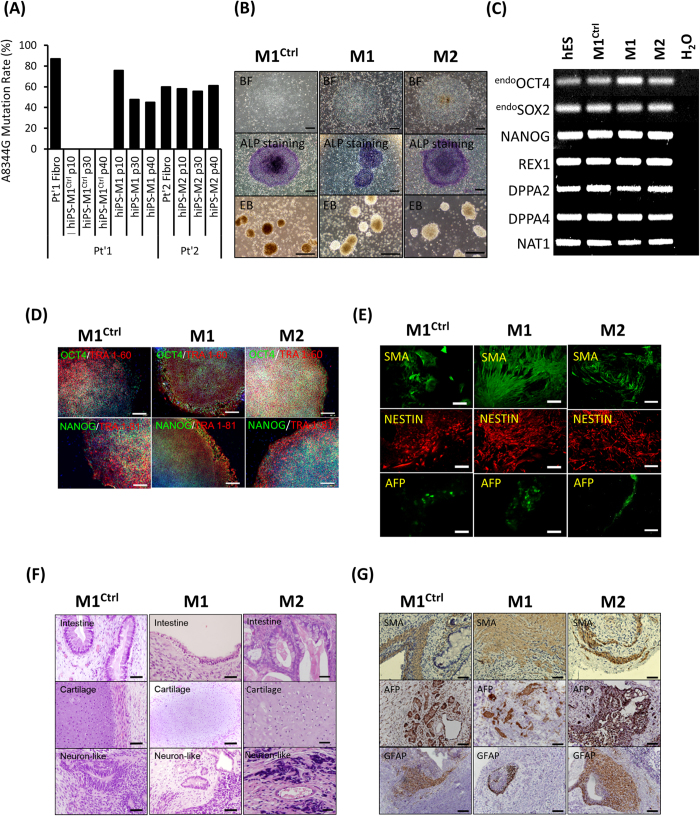
Generation and characterization of hiPSCs harboring mtDNA A8344G. (**A**) The mtDNA 8344 alanine to guanine mutation rate was quantified by pyrosequencing fibroblasts from patients with MERRF syndrome and MERRF fibroblast-derived hiPSCs after indicated passages (p10–40). (**B**) Morphology, alkaline phosphatase activity and embryoid body formation of hiPSCs. Scale bar = 50 μm. (**C**) Reverse transcription polymerase chain reaction (RT-PCR) analysis indicating the expression pattern of embryonic stem cell-like genes in hiPSC sublines. Human embryonic stem cells (hESC) served as a positive control. H2O served as a negative control. (**D**) Immunofluorescence analysis revealing the protein expression of pluripotency markers (OCT4, NANOG, TRA 1–81 and TRA 1–60) in hiPSC sublines. Nuclei were counterstained with Hoechst 33342. Scale bar = 200 μm. (**E**) *In vitro* three-layer differentiation of hiPSCs in specific culture medium showing subpopulations of cells that were immunoreactive for SMA (mesoderm), NESTIN (ectoderm) and AFP (endoderm). Scale bar = 100 μm. (**F**) Sections of teratomas derived from hiPSC sublines showing the differentiation of histologically distinct tissues, including intestine-, cartilage- and neuron-like tissue. Scale bar = 50 μm. (**G**) Immunohistochemistry analysis of sections of teratomas derived from hiPSC subline using SMA (mesoderm), AFP (endoderm) and GFAP (ectoderm). Scale bar = 50 μm.

A mutation at mtDNA A8344G is highly associated with the onset of MERRF syndrome. Specifically, the A8344G mutation blocks the modification of tRNA^Lys^, which impairs the synthesis of mitochondrial proteins that are essential for OXPHOS^8^. However, the underlying mechanism by which the A8344G mutation causes these changes has not been elucidated in a suitable disease model. The disease-specific hiPSCs have been developed as tools to model disease pathogenesis like MERRF syndrome. To generate MERRF-hiPSCs, dermal fibroblasts isolated from the skin biopsies of a MERRF patients (harboring the A8344G mutation) were reprogrammed using retroviruses carrying OCT4, SOX2, KLF4, and GLIS1. Compared to healthy control, cells with A8344G mitochondrial DNA mutation showed dramatically lower reprogramming efficiency (Supp. Fig. S1, n = 3, p < 0.05). It has to been noted that generating hiPSCs from a patient carrying mitochondrial DNA mutations resulted in variant clones, including both mutation frequency-rich and mutation-free clones^19^. To further determine the mutation rate of mtDNA A8344G in the patient-derived fibroblast and MERRF-hiPSC clones, we analyzed the sequence of PCR products amplified from fibroblast and hiPSC mtDNA by pyrosequencing^20^; the amplified and sequencing primers sequences are presented in supplementary table S1. A quantitative analysis showed an approximately 90% mutation rate in fibroblasts and 70% in the 10^th^ passage of hiPSC-M1 derived from patient 1. The mutation rate of hiPSC-M1 further reduced to around 50% in passages later than 30^th^, respectively. In patient M2, the mutation rate is 60% in fibroblast and similar 60% in hiPSC-M2. We also obtained an isogenic mutation-free hiPSC subline (hiPSC-M1^Ctrl^) (Fig. 1A). As expected, these data demonstrate that hiPSCs clones with different mutation frequency can be derived from patients with mtDNA disorders.

To characterize the MERRF-hiPSCs, we analyzed the pluripotency and differentiation capacity of the hiPSC-M1, hiPSC-M2 and hiPSC-M1^Ctrl^ cells. All the hiPSC-M1, hiPSC-M2 and hiPSC-M1^Ctrl^ lines exhibited an embryonic stem cell (ESC)-like morphology and ALP activity (Fig. 1B). Moreover, the expression of stemness genes, including OCT4, SOX2, NANOG, REX1, DPPA2 and DPPA4 was detected by reverse transcription polymerase chain reaction (RT-PCR) analysis (Fig. 1C), and the protein expressions of OCT4, NANOG, TRA1-60 and TRA 1-81 were confirmed by immunofluorescence staining (Fig. 1D). Furthermore, we examined the *in vitro* differentiation ability of MERRF hiPSCs. A suspension culture was prepared to allow the formation embryoid bodies (EB, Fig. 1B), which were then seeded in 0.1% gelatin-coated chamber-slide wells with differentiation medium. After 2 weeks of induction, immunofluorescence detected differentiated cells positive for alpha smooth muscle actin (α-SMA, a marker of mesoderm), nestin (a marker of the ectoderm) and alpha fetal protein (AFP, a marker of the endoderm) (Fig. 1E). To further assess pluripotency *in vivo*, we injected MERRF hiPSCs (M1, M2 and M1^Ctrl^) into the testicles of severe combined immunodeficiency (SCID) mice. Teratoma formation was first observed 12 weeks after hiPSCs injection. Histological examination revealed that the teratoma consisted of three embryonic germ layers, including intestine-like tissues (endoderm), cartilage (mesoderm), and neuron-like tissues (ectoderm) (Fig. 1F). The teratoma also stained with α-SMA, AFP and GFAP (a marker of the ectoderm), respectively, to further demonstrated the differentiation ability (Fig. 1G). The hiPSC-M1, hiPSC-M2 and hiPSC-M1^Ctrl^ exhibited pluripotency and the ability to differentiate into three germ layers. Collectively, our results demonstrated that hiPSC-M1, hiPSC-M2 and isogenic hiPSC-M1^Ctrl^ have similar capablility of differentiation.

### mtDNA A8344G impairs mitochondrial function, increases ROS production and decreases cell proliferation

**Figure 2:**
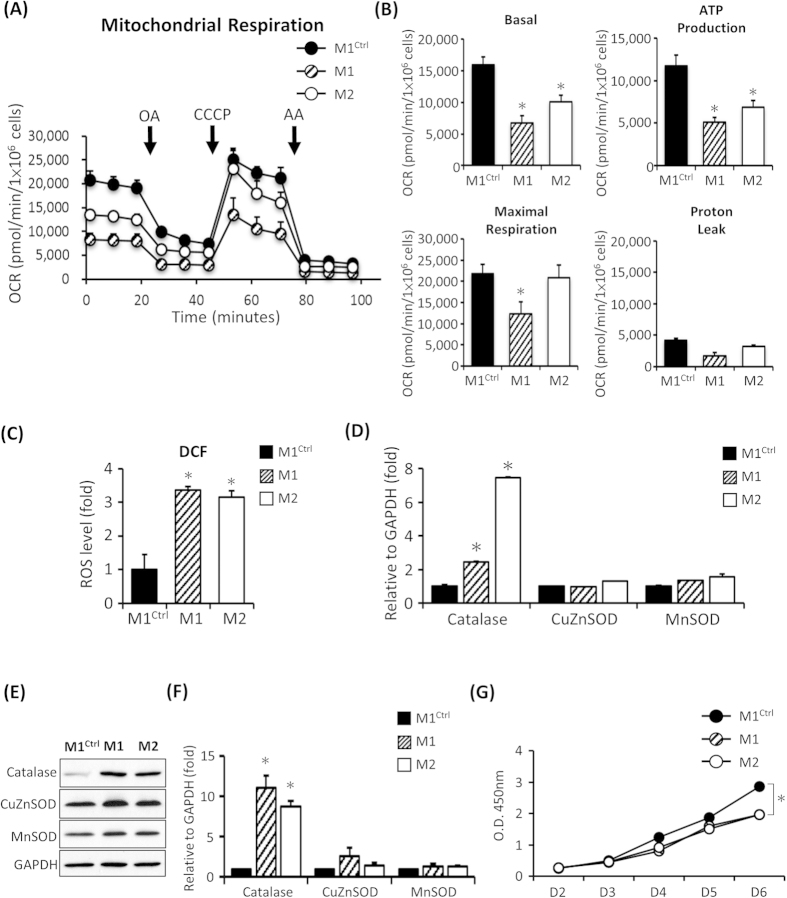
Impaired mitochondrial functions and accumulation of endogenous ROS in MERRF-specific hiPSCs harboring mtDNA A8344G. (**A**) Representative data showing the oxygen consumption rate (OCR) in M1, M2 and M1^Ctrl^ hiPSC sublines. (**B**) Quantitative data of basal oxygen consumption, ATP production, maximal respiration and proton leak. Data are the mean ± SEM, n = 3. (**C**) Intracellular ROS production of hiPSC as indicated by DCFH-dA staining; fluorescence intensity was measured by flow cytometry. The intensity of the M1^ctrl^ line was defined as 100%. Data are the mean ± SEM, n = 3. (**D**) qPCR demonstrating the expression level of antioxidant-associated enzymes. Data are the mean ± SEM, n = 3 (**E**) Western blot indicated the antioxidant enzymes expression. (**F**) The data represented mean ± SEM of (**E**), n = 3. (**G**) Cell proliferation rate of hiPSC sublines. Cell proliferation was determined based on the CCK-8 kit. M1 and M2, MERRF induced pluripotent stem cells. M1^Ctrl^, MERRF induced pluripotent stem cells carrying normal mtDNA (A8344G free subline).

It’s been reported that mtDNA A88334G mutation can alter mitochondrial function and decrease ATP synthesis in fibroblasts derived from patients with MERRF syndrome^21^,^22^. Therefore, we used the extracellular flux analyzer to measure oxygen consumption of mitochondria to evaluate the function of the respiratory chain. To assess the function of the electron transport chain (ETC), we added oligomycin A, CCCP and antimycin A, which uncouples ATP synthase from cytochrome *b* to cytochrome *c1* and inhibits the ETC (Fig. 2A). The basal oxygen consumption rate (OCR) of the MERRF-hiPSCs (M1 and M2) were reduced compared with the hiPS-M1^Ctrl^ (Fig. 2B, n = 3, p < 0.05). Moreover, the ATP production rate and maximal respiration were also reduced in the hiPSC-M1 and M2 (Fig. 2B, n = 3, p < 0.05). This oxygen consumption defect may be due to either impaired ETC function or an uncoupling of the ETC from ATP synthase activity. The extracellular flux analyses demonstrated that the hiPSC-M1 and M2 exhibited defect OCR due to reduced ETC function.

Mitochondrial dysfunction may often endogenously elevate ROS levels and alter the expression of antioxidant genes. In fact, previous studies showed that excessive mtDNA mutation-elicited oxidative stress and oxidative damage were observed in patients with MERRF syndrome^8^. To further determine the level of intracellular ROS, we stained hiPSCs with DCFH-dA. Intracellular ROS contents of the hiPSC-M1 and hiPSC-M2 were significantly higher than those of the hiPSC-M1^Ctrl^ (Fig. 2C, n = 3, p < 0.05). We hypothesized that elevated ROS production correlates with impaired antioxidant expression. Therefore, we analyzed the expression of antioxidant gene including catalase, CuZnSOD and MnSOD using quantitative reverse transcription polymerase chain reaction (q-PCR). Intriguingly, the results showed elevated catalase expression in the hiPSC-M1 and hiPSC-M2, but the levels of MsSOD and CuZnSOD remained the same as hiPSC-M1^Ctrl^ (Fig. 2D, n = 3, p < 0.05). Furthermore, the protein level of catalase also increased as shown in Western blot analysis (Fig. 2E). The quantified data from Fig. 2E indicated that protein expression of catalase was significantly increased in MERRF-hiPSCs compared to isogenic control M1^Ctrl^ (Fig. 2F). Taken together, using hiPSCs as an *in vitro* MERRF disease model, our results indicated that the intra-cellular ROS levels and expression of the antioxidant gene catalase were significantly increased in the hiPS-M1 compared with the isogenic hiPSC-M1^Ctrl^ in the pluripotent state.

Furthermore, fibroblasts derived from patients with MERRF syndrome and depolarized embryonic stem cells due to CCCP treatment showed reduced proliferation^23^,^24^. To determine the proliferation rate of MERRF hiPSCs, we used cell counting kit 8 (CCK-8) to assess the cell viability and estimate cell growth. Cell proliferation was significantly reduced in hiPSCs carrying the mtDNA A8344G mutation (hiPSC-M1 and hiPSC-M2) compared with the isogenic hiPSC-M1^Ctrl^ (Fig. 2G, n = 3, p < 0.05). These data suggest that impaired mitochondria can cause reduced proliferation in hiPSCs.

### Fragmented mitochondrial morphology in MERRF hiPSCs

**Figure 3:**
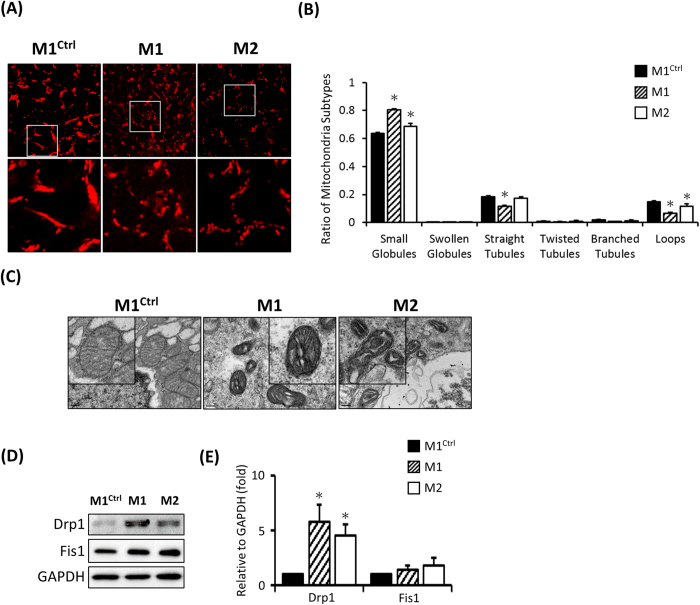
Detection of mitochondrial distribution and morphology in MERRF hiPSCs. (**A**) Mitochondrial distribution stained by MitoTracker Red and analyzed by confocal microscopy. (**B**) Quantitative data revealing mitochondrial subtypes based on a microP analysis. (**C**) Ultra-structure of hiPSC-M1, hiPSC-M2 and hiPS-M1^Ctrl^ by TEM. (**D**) Western blot showing the expression of mitochondrial fission proteins. (**E**) Quantitative result of mitochondrial fission proteins, the data represented mean ± SEM of (**D**), n = 3. M1 and M2, MERRF induced pluripotent stem cells. M1^Ctrl^, MERRF induced pluripotent stem cells carrying normal mtDNA (A8344G free subline).

An image analysis of MERRF-derived fibroblast based on MitoTracker staining indicated small, rounded, depolarized mitochondria^23^. Accordingly, to determine mitochondria morphology, we stained hiPSCs with MitoTracker Red. Smaller, rounded, and depolarized mitochondria were noted in the hiPSC-M1 and M2 compared with hiPSC-M1^Ctrl^ (Fig. 3A). To further evaluate the mitochondrial subtypes, we analyzed mitochondria with MicroP software, which is an automated tool used to quantify mitochondrial morphology^25^. This analysis showed more small globular mitochondria in hiPSC-M1 and M2 than in hiPSC-M1^Ctrl^. Conversely, hiPSC-M1^Ctrl^ exhibited mitochondria characterized by continuous tubules and loops (Fig. 3B, n = 200, p < 0.05). Furthermore, transmission electron microscopy (TEM) analysis revealed fragmented mitochondria in hiPS-M1 and M2 (Fig. 3C). Since mitochondria are dynamic organelles influenced by fusion and fission proteins, we therefore assessed the involvement of fission proteins via Western blot. Drp1 expression was increased in hiPSC-M1 and hiPSC-M2, suggesting that the fragmented mitochondria were dissociated by Drp1 (Fig. 3D,E). Collectively, these data indicate that the mitochondria were small, globular and fragmented in A8344G mutation-harboring hiPSC-M1 and hiPSC-M2.

### Low mutation rate in MERRF hiPSC-derived cardiomyocytes

**Figure 4:**
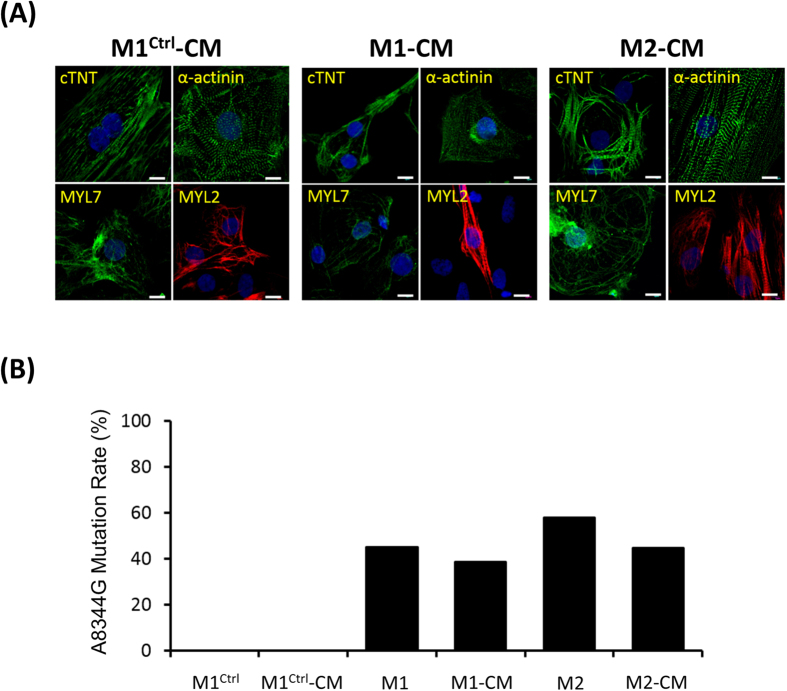
Characterization of MERRF hiPSC-derived cardiomyocytes (MERRF hiPSC-CMs). (**A**) Immunofluorescence of MERRF hiPSC-CMs stained with cardiomyocyte markers, including cTNT, α-actinin, MYL7 and MYL2. Nuclei were counterstained with Hoechst 33342 (blue). Scale bar, 10 μm. (**B**) mtDNA A8344G mutation rate quantified by pyrosequencing of MERRF hiPSCs and MERRF hiPSC-derived cardiomyocytes. M1-CM and M2-CM, MERRF hiPSC-derived cardiomyocytes carried mtDNA A8344G mutation. M1^ctrl^-CM, hiPSC-derived cardiomyocyte carried normal mtDNA.

Cardiomyocytes (CMs) require high levels of mitochondria to support high levels of energy consumption, and over half of the patients with MERRF syndrome displayed cardiomyopathy^26^. To evaluate the pathological effects of MERRF in cardiomyocyte, we investigated the induction ability and mitochondrial functions in cardiomyocyte-like cells differentiated from MERRF-hiPSCs. Two weeks of induction in a cardiac differentiation medium resulted in spontaneously-beating cardiomyocyte-like cells. Immunofluorescent staining further confirmed the expression of cardiac-specific markers, including cardiac troponin T (cTNT), α-actinin, myosin light chain 7 (MYL7), and MYL2 (Fig. 4A). Next, we used pyrosequencing to determine the mtDNA A8344G mutation rate. This analysis showed a similar mutation rate of hiPSC-M1 and hiPSC-M2-derived cardiomyocytes (M1-CMs and M2-CMs, whereas cardiomyocytes differentiated from the hiPSC-M1^Ctrl^ (M1^Ctrl^-CMs) remained free of mutation (Fig. 4B). These data suggest that MERRF hiPSCs continued to exhibit multi-lineage differentiation ability, including cardiogenic differentiation.

### Impaired mitochondrial function and increased ROS production in MERRF hiPSC-derived cardiomyocytes

**Figure 5:**
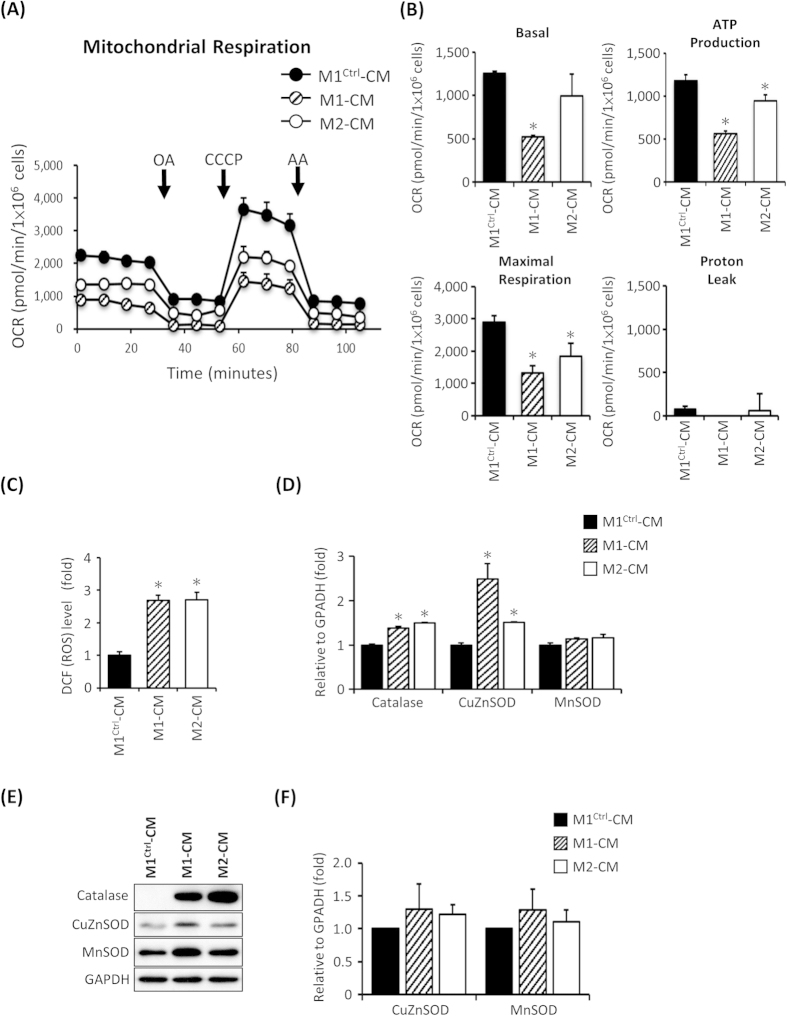
Oxygen consumption of mitochondria as detected with an extracellular flux assay and accumulation of endogenous ROS and altered antioxidant enzyme expression in hiPSC-derived cardiomyocytes. (**A**) Representative data showing the OCR of M1-CM, M2-CM and M1^Ctrl^-CM. (**B**) Total basal oxygen consumption, ATP production, maximal respiration and proton leak data. Data are the mean ± SEM, n = 3. (**C**) Intracellular ROS production of MERRF-hiPS-derived cardiomyocytes was stained using DCFH-dA. The staining of M1^Ctrl^-CM was defined as 100%. Data are the mean ± SEM, n = 3. (**D**) qPCR revealing the antioxidant-associated enzyme expression level in hiPSC-CMs. Data are the mean ± SEM, n = 3. (**E**) Western blot showing the expression of mitochondrial antioxidant enzyme. (**F**) Quantitative result of antioxidant proteins, the data represented mean ± SEM of (**E**), n = 3. M1-CM and M2-CM, MERRF hiPSC-derived cardiomyocytes carried mtDNA A8344G mutation. M1^ctrl^-CM, hiPSC-derived cardiomyocyte carried normal mtDNA.

In addition, an extracellular flux analyzer was performed to further estimate the OCR of mitochondrial respiration and assess the mitochondrial functions of hiPSC derived-CMs. This assay indicated that M1-CMs and M2-CMs displayed minor decreases in oxygen consumption as compared with M1^Ctrl^-CMs (Fig. 5A, n = 3). Figure 5B presents a quantitative analysis of the basal, ATP-couple, maximal and proton leak –associated OCR, respectively. These findings confirm that the mtDNA A8344G mutation results in impaired mitochondrial respiration in MERRF hiPSC-CMs.

Next, we stained MERRF hiPSC-CMs with DCFH-dA to detect the intracellular ROS production. The results indicated higher ROS levels in M1-CMs and M2-CMs compared with M1^Ctrl^-CMs (Fig. 5C, n = 3, p < 0.05). Furthermore, qRT-PCR showed increased expression of antioxidant enzymes, including catalase and CuZnSOD (Fig. 5D, n = 3, p < 0.05). Western blot also indicated that catalase expression was increased in M1-CMs and M2-CMs compared with M1^Ctrl^-CMs (Fig. 5E). The quantified data from Fig. 5E indicated that protein expression of CuZnSOD and MnSOD was no significant difference in MERRF-hiPSCs compared to isogenic control M1^Ctrl^ (Fig. 5F). Collectively, using MERRF hiPSC-CMs as an *in vitro* disease model, our results indicate that the cellular ROS levels and expression of the ROS-associated gene catalase were significantly increased in M1-CMs and M2-CMs compared with the mutation-free M1^Ctrl^-CMs.

### Fragmented mitochondrial morphology in MERRF hiPSC-derived CM

**Figure 6:**
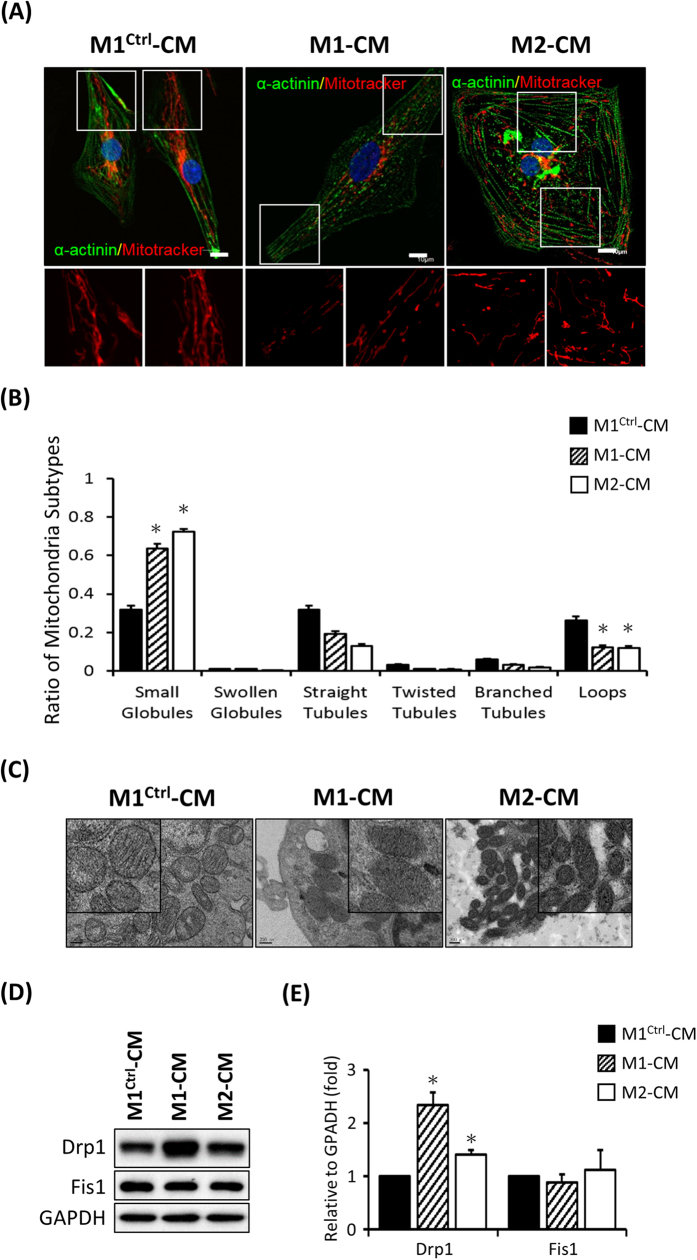
Fragmented mitochondrial morphology in MERRF-hiPSC-derived cardiomyocytes. (**A**) Mitochondria were stained with MitoTracker Red, and immunofluorescence analysis indicated the cardiomyocyte marker α-actinin in MERRF hiPSC-derived cardiomyocytes. (**B**) Quantitative data showing mitochondrial subtypes based on a microP analysis. (**C**) Ultra-structure of M1-CM, M2-CM and M1^Ctrl^–CM. (**D**) Western blot revealing the expression of the mitochondrial fission proteins. (**E**) Quantitative result of mitochondrial fission proteins, the data represented mean ± SEM, n = 3. M1-CM and M2-CM, MERRF hiPSC-derived cardiomyocytes carried mtDNA A8344G mutation. M1^ctrl^-CM, hiPSC-derived cardiomyocyte carried normal mtDNA.

Moreover, to determine the mitochondrial integrity, we stained cells with MitoTracker Red. It’s found that mitochondria were fragmented in M1-CMs and M2-CMs but not in M1^Ctrl^-CMs (Fig. 6A). Furthermore, an image analysis by MicroP also revealed more small globular mitochondria in M1-CMs and M2-CMs (Fig. 6B, n = 50, p < 0.05). Next, the ultrastructure of mitochondria was observed by TEM, and numerous degenerative mitochondria were found in M1-CMs and M2-CMs (Fig. 6C). Western blots also demonstrated increased expression of fission protein Drp1 in M1-CMs and M2-CMs (Fig. 6D,E). Taken together, our data demonstrated that MERRF hiPSC-derived CMs contained numerous degenerative and fragmented mitochondria accompany with severe mitochondrial dysfunction, suggesting that MERRF hiPSC system served as an *in vitro* pathophysiological model for mimicking MERRF disease.

### Impaired mitochondrial function, increased ROS production and fragmented mitochondria in MERRF hiPSC-derived NPC

**Figure 7:**
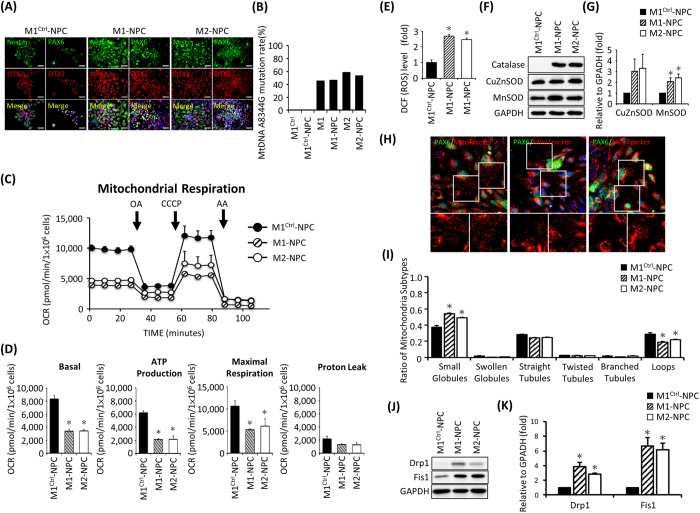
Oxygen consumption of mitochondria as detected with an extracellular flux assay and accumulation of endogenous ROS and altered antioxidant enzyme expression in hiPSC-derived neural progenitor cells. (**A**) Immunofluorescence of MERRF hiPSC-NPCs stained with neural markers Nestin, PAX6 and OTX2. Nuclei were counterstained with Hoechst 33342 (blue). Scale bar = 50 μm. (**B**) mtDNA A8344G mutation rate quantified by pyrosequencing of MERRF hiPSCs and MERRF hiPSC-derived neural progenitor cells. (**C**) Representative data showing the OCR of hiPSC-M1-, M2- and M1^Ctrl^-derived NPC. (**D**) Total basal oxygen consumption, ATP production, maximal respiration and proton leak data. Data are the mean ± SEM, n = 3. (**E**) Intracellular ROS production of MERRF-hiPS-derived NPC was stained using DCFH-dA. The staining of M1^Ctrl^-NPC was defined as 100%. Data are the mean ± SEM, n = 3. (**F**) Western blot showing the expression of antioxidant proteins. (**G**) Quantitative result of antioxidant proteins. The data represented mean ± SEM, n = 3. (**H**) MitoTracker Red stained the mitochondria of MERRF hiPSC-NPCs. PAX6 served as a neural marker. (**I**) Quantitative data showing mitochondrial subtypes based on a microP analysis. (**J**) Western blot showing the expression of mitochondrial fission proteins. (**K**) Quantitative result fission proteins. The data represented mean ± SEM, n = 3. M1-NPC and M2-NPC, MERRF hiPSC-derived neural progenitor cells carried mtDNA A8344G mutation. M1^ctrl^-NPC, hiPSC-derived neural progenitor cells carried normal mtDNA.

MERRF syndrome is clinically characterized by generalized epilepsy, cerebellar ataxia, deafness, dementia and hereditary polyneuropathies^27^. To further evaluate the multiorgan failure of MERRF in neural cells, we investigated the mitochondrial functions in neural progenitor cells differentiated from MERRF-hiPSCs (M1- and M2-NPCs). Immunofluorescent staining was performed to confirm neural differentiation of hiPSCs with the neural progenitor markers Nestin, PAX6 and OTX2 (Fig. 7A). Next, mitochondrial DNA A8344G mutation rate in M1-NPCs and M2-NPC were confirmed by pyrosequencing (Fig. 7B). Mitochondrial functions of MERRF hiPSC-NPCs were performed and the data indicated that M1-NPCs and M2-NPCs displayed decreased basal oxygen consumption, ATP production and maximal respiration (Fig. 7C,D, N = 3, p < 0.05). These findings further confirmed that the mtDNA A8344G mutation resulted in impaired mitochondrial respiration.

Further, we stained MERRF hiPSC-NPC with DCFH-dA to detect the intracellular ROS production. This staining indicated higher ROS levels in M1-NPCs and M2-NPCs compared with M1^Ctrl^-NPCs (Fig. 7E, n = 3, p < 0.05). Western blot showed increased expression of catalase and MnSOD (Fig. 7F,G, n = 3, p < 0.05). MERRF-hiPSC-NPCs stained with MitoTracker Red showed fragmented mitochondria but not in M1^Ctrl^-NPCs (Fig. 7H,I, n = 50, p < 0.05). The expression of fission protein Drp1 and Fis1 increased in M1-NPCs and M2-NPCs (Fig. 7J,K). Taken together, our data demonstrated that MERRF hiPSC-derived NPCs contained numerous degenerative and fragmented mitochondria accompany with increased ROS and mitochondrial dysfunction which support the multiorgan effect of MERRF syndrome.

## Discussion

MERRF syndrome is one of the mitochondrial encephalomyopathies and the clinical phenotypes and mechanism of pathogenesis of this disease has been studied for more than three decades. However, a lack of fundamental knowledge regarding the biology and genetics of the mitochondrion limits a deeper understanding of the inheritance and pathophysiology of mitochondrial diseases. In the present study, we establish hiPSCs from patients with MERRF syndrome carrying the mtDNA A8344G mutation. MERRF hiPSCs exhibited reduced growth and impaired mitochondrial function. Heart and cardiomyocytes require a high level of mitochondria to support their high energy demand, and patients with MERRF syndrome consequently exhibit cardiomyopathy. Impaired mitochondria are associated with elevated intracellular ROS production and altered antioxidant gene expression in both hiPSCs and hiPSC-derived cardiomyocytes. Specifically, mitochondria appear to be fragmented in MERRF-derived cardiomyocytes. Collectively, this report represents the first study using MERRF hiPSCs as well as MERRF hiPSC-CMs to demonstrate the pathophysiology of mtDNA A8344G mutation.

Notably, in the present study, we obtained both mutation-rich and mutation-free hiPSCs from the same donor. The mutation rate of mtDNA is the result of a dynamic distribution during reprogramming and culture. In 2012, Fujikura *et al.* obtained mutation-rich and mutation-free hiPSCs from diabetic patients harboring the mtDNA A3243G mutation^28^. Similar to our finding, mtDNA heteroplasmy, a phenomenon of more than one type of mtDNA genome, was also observed in Pearson marrow pancreas syndrome (PS)-iPSC^19^. The mechanism that causes variant mtDNA heteroplasmy remains unclear but has been suggested to be related to the mitochondria bottleneck effect. Furthermore, mtDNA dynamics during cardiac differentiation were not observed in the present study, but the outcome of mitochondrial dysfunction in cardiomyocytes was evaluated. Taking our findings together, we generated a hiPSC model of mutation-rich and mutation-free cells that share the same genetic background. This model is useful to clarify the pathophysiology and biomolecular mechanisms of MERRF syndrome.

Increased ROS levels and oxidative stress are common consequences of mitochondrial dysfunction, and previous studies have described increased ROS levels in fibroblasts and cybrids carrying mtDNA A8344G mutation^8^,^23^. Specifically, impaired mitochondria result in increased ROS levels and influence antioxidant gene expression^7^,^29^. Our previous studies also showed that ROS alter the expression of prohibitin, Lon protease, aconitase and matrix metalloproteinase 1 (MMP1) in the skin fibroblasts of the MERRF patients^8^,^30^. Furthermore, co-enzyme Q10 supplementation eliminates mitochondrial dysfunction and decreases ROS levels in fibroblasts and hybrid clones containing A8344G mutation^23^.

Impaired mitochondria not only increase the level of ROS but also decrease the production of ATP. The mitochondrial content and mtDNA copy number decrease after cellular reprogramming^31^,^32^. However, the functions of mitochondria normalized to mitochondrial mass are similar to those of somatic cells^33^. A previous study demonstrated that mitochondria function regulates cell proliferation and early differentiation potential of ESC. The inhibition of mitochondrial oxidative phosphorylation results in impaired ESC proliferation and altered early differentiation^24^. The growth rate of MERRF hiPSCs were reduced in present study, indicating that impaired mitochondrial function may prevent stem cells from maintaining its population. Besides, lower reprogramming rates were also observed in fibroblast derived from patients carried with mtDNA A8344G mutation (Supp. Fig. S1). We did not rule out the possibility that the mtDNA A8344G may alter the pluripotency and differentiation potential of MERRF hiPSCs.

To date, studies of MERRF syndrome have relied on patient-derived fibroblast and cybrid clone models, which do not fully represent the physiological symptoms of MERRF. Moreover, the additional features of MERRF include epilepsy, ataxia, peripheral neuropathy, dementia and cardiomyopathy. Patients with this condition may also develop hearing loss or optic atrophy, which is the degeneration of nerve cells that carry visual information from the eyes to the brain^27^. In our study, we differentiated MERRF-iPSCs into cardiomyocytes and neural progenitor cells that exhibited elevated ROS levels and impaired mitochondrial function. Furthermore, iPSCs may serve as powerful tools for disease models because they can differentiate into neurons or cells of the optic cup, which facilitates the study of hearing loss and visual problems, respectively.

**Figure 8:**
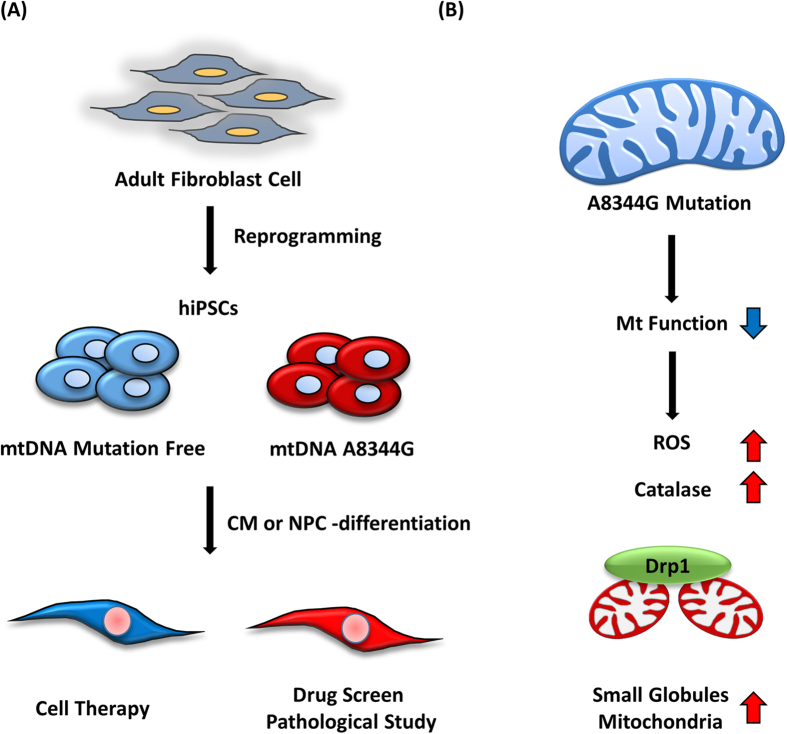
MERRF-specific hiPSCs as an *in vitro* disease model. (**A**) Generation of hiPSC lacking the mitochondrial DNA mutation created mutation-free and mutation-rich clones. The mutation-free clones may be useful for cell therapy and the MERRF-specific hiPSC with the mutation rich clones are suitable for drug screening and pathological research. (**B**) The mtDNA A8344G mutation impairs mitochondrial function and results in the accumulation of endogenous ROS and fragmented mitochondrial morphology.

In conclusion, we generated mutation-free hiPSCs and hiPSCs harboring mtDNA mutations. The mutation-free clones may serve as healthy material for cell therapy, while the mutation-rich clones are suitable for drug screening and pathological studies (Fig. 8A). Furthermore, both hiPSCs and hiPSCs-differentiated CMs and NPCs that harbor the mtDNA A8344G mutation exhibit impaired mitochondrial function, elevated intracellular ROS levels, and fragmented mitochondrial morphology (Fig. 8B). This study established a hiPSC model of MERRF syndrome to assess mitochondrial dysfunction. Although the underlying mechanism of A8344G mutation-derived mitochondrial dysfunction remains unclear, iPSCs generated from patients with MERRF syndrome represent useful models for the exploration of disease pathogenesis and pharmacologic screening. Future studies are needed to define the effect of impaired mitochondria on the ROS levels and the relationship between the biomolecular mechanism for ROS over-production and MERRF-derived cardiomyopathy.

## Material and Method

### Patients’ information and cell culture

This research followed the tenets of the Declaration of Helsinki, and all samples were obtained after patients had given informed consent. The protocols for this study were approved by the by the Internal Research Board of Mackay Medical College and Taipei Veterans General Hospital. The patients in this study were a 15-year-old Chinese female (M1) had poor learning, myoclonus and myoclonic epilepsy and her 13-year-old sister (M2)^34^. Human fibroblast cells were cultured in Dulbecco’s modified Eagle medium (DMEM) containing 10% fetal bovine serum (FBS, Hyclone), nonessential amino acids, sodium pyruvate and penicillin/streptomycin. Human embryonic stem cells and induced pluripotent cells were cultured in hESCs medium, which consisted of DMEM/F12 supplemented with 20% knockout serum replacement (KSR), nonessential amino acids, L-glutamine, penicillin/streptomycin, 0.1 mM β-mercaptoethanol and 10 ng/ml basic fibroblast growth factor (bFGF). The medium was changed every day. ESCs and iPSCs were maintained on mitomycin C-inactivated mouse embryonic fibroblasts and passaged every 5 to 7 days using collagenase IV. Feeder-free culture conditions were applied, which consisted of mTeSR™1 medium (Stemcell Technologies) and coating by Geltrex (Life Technologies).

### Generation of human induced pluripotent stem cells (hiPSCs)

Human iPSCs were generated from human fibroblasts derived from donors with MERRF syndrome. The iPSCs were reprogrammed by the transduction of retroviral vectors encoding four transcription factors, as described previously^35^. Briefly, the plasmids pMXs-OCT4, SOX2, KLF4, and GLIS1 (Addgene) were individually packaged into retroviral particles by transfection into 293 T cells using the TransIT-LT1 (Mirus). Retroviral transduction was performed two times at one-day intervals. After 1 week of transduction, 1 × 10^5^ infected fibroblasts were re-seeded on inactivated MEF feeder cells. The following day, the medium was replaced with hESCs medium and changed every day. After 21 to 28 days of re-seeding, the colonies were each transferred to feeder cultures in organ culture dishes (ODC; BD) to develop additional colonies for characterization.

### mtDNA A8344G mutation rate assay

Pyrosequencing was performed by Misson Biotech Corporation and according to HELEN E. WHITE *et al.* protocol^20^. In brief, the biotin-labeled PCR products were captured by Streptavidin-Sepharose HP (Amersham Pharmacia). PCR products bound on the bead were purified and made single stranded using a Pyrosequencing Vacuum Prep Tool. The sequencing primers were annealed to the single-stranded PCR product, and pyrosequencing was done using the PyroMark Q24 system (Qiagen). Quantitation of cytosine methylation was done using the PyroMark Q24 software. The required primers (mtF8203, mtR8394 and Sequencing) were listed in supplementary table S1.

### Alkaline phosphatase staining and immunofluorescence staining

Alkaline phosphatase (ALP) staining was performed using the Blue Alkaline Phosphatase Substrate Kit (Vector) following the manufacturer’s instructions. Immunofluorescence staining was performed with the indicated antibodies. Briefly, cells were sub-cultured in 4-well chamber-slides (Millipore). The cells were fixed in 1% (w/v) paraformaldehyde for 10 minutes at RT and washed thrice with phosphate-buffered saline (PBS). An additional permeabilization step (1% (v/v) NP-40 in PBS) was performed prior to staining with antibodies for internal cell markers. Non-specific staining was blocked by incubation in 3% (w/v) bovine serum albumin (Sigma) and 5% (v/v) FBS. The cells were incubated with primary antibodies for 16 hours at 4 °C. Following this incubation, cells were washed thrice with PBS and then incubated with the corresponding secondary antibodies (1:200) for 2 hours at RT, which were purchased from Jackson ImmunoResearch Laboratories. The primary antibodies used in this study include anti-OCT4 (1:100; Cell Signaling), anti-NANOG (1:100; Cell Signaling), anti-TRA 1–60 (1:100; CHEMICON), anti-TRA 1–81 (1:100; Cell Signaling), anti-alpha fetoprotein (AFP) (1:100; Cell Signaling), anti-Nestin (1:100; Millipore), anti-alpha-smooth muscle actin (SMA) (1:200; Biomeda) and anti-neurofilament (NF; 70 kDa; 1:100; Millipore).

### RT-PCR and quantitative-PCR

Total RNA (5 μg) was purified with TRIzol reagent (Life technologies), and cDNA was synthesized using SuperScript Reverse Transcriptase III (Life technologies) according to manufacturer’s instructions. PCR was performed using the Taq DNA Polymerase Master Mix (Ampliqon). Quantitative PCR was performed using SYBR-Green PCR master mix and an ABI-7900 real-time analyzer. The gene-specific primer sequences are presented in supplementary table S2.

### Embryoid body formation and *in vitro* differentiation

The iPSCs were detached by collagenase IV treatment and plated onto ultra-low attachment dishes in hESCs medium without bFGF. After 3 days, the EBs were collected and seeded in 0.1% gelatin-coated 4-well chamber-slides in differentiation medium. After 2 weeks of differentiation, the cells were collected for further analysis.

### Teratoma formation, H&E staining and IHC

When the iPSCs reached 80% confluence 6 cm dishes, they were harvested by collagenase IV treatment, collected into tubes and injected below the capsule of the testes of 6- to 12-week-old SCID mice. After 9 to 12 weeks of injection, the teratomas were collected and fixed with 4% paraformaldehyde. The paraffin-embedded tissue was sectioned and stained with hematoxylin and eosin (H&E). The paraffin embedded sections were deparaffinized and rehydrated in Target Retrieval Solution (DaKo). Sections were blocked with 3% fetal bovine serum for 5 mins and incubated with the primary antibodies for 30 mins at room temperature. The primary antibodies used in this assay were anti-AFP (1:100; Cell Signaling), anti-GFAP (1:300; Cell Signaling), anti-SMA (1:100; Abcam).

### Cell proliferation assay

Cell counting kit 8 (CCK-8, Dojindo) was applied for cell proliferation assay to determine of cell viability following manufacturer’s protocol. In Brief, cells were seeded in 24-well plates at concentration of 1 × 10^4^ cells per well and cultured for 2 days. CCK-8 solution were added into plates and incubated for 4 hours. The supernatant was assayed by measuring the absorbance at 450 nm.

### Extracellular flux assay

The cells were dissociated into single cells by Versene treatment (Gibco) at 37 °C for 4 to 5 min and seeded onto geltrex-coated XF24 cell culture plate at 5 × 10^4^ cells per well with 10 μM Y-27632. One day later, the oxygen consumption rates of the cells were measured using an XF24 Extracellular Flux Analyzer in unbuffered medium to assess ATP production, spare respiratory capacity and proton leakage. Three mitochondrial inhibitors, oligomycin, CCCP and antimycin A, were sequentially injected during the measurements to determine the mitochondrial function. The results were normalized to the cell number and analyzed by using the Seahorse XF24 software.

### Measurement of reactive oxygen species

The intracellular ROS level was measured by staining with 5 μM DCFH-dA at 37 °C for 30 min. The fluorescence intensity of 1 × 10^5^ cells was analyzed using the BD FACS Calibur flow cytometer.

### Cardiac differentiation

Cardiac differentiation was induced in RPMI/B27 medium with Wnt/β-catenin inhibitors, as described previously^36^. Briefly, cells were dissociated by Versene (Life technologies) and seeded onto Geltrex-coated plates at a density of 3 × 10^5^ cells/cm^2^. On day 0, the cells were treated with 6 μM CHIR99021 in insulin-free RPMI/B27 medium for 24 hours. The medium was replaced with basal medium for another 2 days. On day 3, the culture medium was replaced with 5 μM IWP2 in insulin-free RPMI/B27 for 48 hours. On day 7, the culture medium was changed to RPMI/B27 containing insulin, and the culture medium was refreshed thereafter every 3 days.

### MitoTracker Red staining and immunostaining

The integrity of the mitochondrial membrane was tested using the fluorescent dye MitoTracker (Life Technologies). Briefly, live cells growing on 60-μm dishes (ibidi) were incubated in media containing 200 nM MitoTracker Red for 15 min at 37 °C in a 5% CO_2_ incubator. The cells were then fixed with 1% paraformaldehyde. The fixed cells were permeabilized with 1% (v/v) NP-40 in PBS before staining with antibodies for internal cell markers. The primary antibodies used in this assay were anti-α-actinin (1:200; Cell Signaling), anti-Troponin T (cTNT, 1:200; Millipore), anti-MYL2 (1:200; Millipore) and anti-MYL7 (1:200; Millipore). Confocal microscopic images were then obtained using a Zeiss Confocal Fluorescence Microscope (LSM 510 META, Version 3.2 SP2, Carl Zeiss).

### Transmission electronic microscope

The cells were fixed in 4% paraformaldehyde, 2% glutaraldehyde and 0.1 M cacodylate at 4 °C overnight and then embedded in epoxy resin and sectioned. Ultrathin sections were counterstained with 2.5% uranyl acetate and 0.4% lead citrate and then viewed with a JEM-2000EXII electron microscope.

### Statistics

The results are presented as the mean ± standard error of the mean (SEM). The ROS fluorescence intensity, real-time qPCR and OCR data were compared using the student’s t-test. All histograms and images were generated using Excel 2013. P-values less than 0.05 were considered significant.

## Additional Information

**How to cite this article**: Chou, S.-J. *et al.* Impaired ROS Scavenging System in Human Induced Pluripotent Stem Cells Generated from Patients with MERRF Syndrome. *Sci. Rep.* **6**, 23661; doi: 10.1038/srep23661 (2016).

## Supplementary Material

Supplementary Information
