# Supplementary material for: Impaired ROS Scavenging System in Human Induced Pluripotent Stem Cells Generated from Patients with MERRF Syndrome
Source: Sci Rep. 2016 Mar 30;6:23661. doi: 10.1038/srep23661 (PMC4812254; doi:10.1038/srep23661)
Supplement: Supplementary Information [file srep23661-s1.pdf]

Impaired ROS Scavenging System in Human Induced Pluripotent Stem Cells Generated from Patients with MERRF Syndrome

Shih-Jie Chou[1], Wei-Lien Tseng[1,5], Chien-Tsun Chen[4], Yu-Fen Lai[2,5], Chian-Shiu Chien[3,5], Yuh-Lih Chang[1,5], Hsin-Chen Lee[1,3]*, Yau-Huei Wei[4]*, Shih-Hwa Chiou[1,2,3,5]*

[1] Institute of Pharmacology, [2] Institute of Clinical Medicine, [3] School of Medicine, National Yang-Ming University, Taipei, Taiwan; [4] Department of Medicine, Mackay Medical College, New Taipei, Taiwan; [5] Department of Medical Research, Taipei Veterans General Hospital, Taipei, Taiwan

***Correspondence:**

Hsin-Chen Lee, PhD
Institute of Pharmacology, National Yang-Ming University, Taipei, Taiwan
E-mail: hclee2@ym.edu.tw

Yau-Huei Wei, PhD
Department of Medicine, Mackay Medical College, New Taipei, Taiwan
E-mail: joeman@mmc.edu.tw

Shih-Hwa Chiou, MD, PhD
Department of Medical Research, Taipei Veterans General Hospital
Institute of Pharmacology, National Yang-Ming University
No. 201, Sec.2 Shih-Pai Road, Taipei, Taiwan.
Tel: 886-2-28757394
Fax: 886-2-28757435
E-mail: shchiou@vghtpe.gov.tw

Supplementary Figure S1

(A)

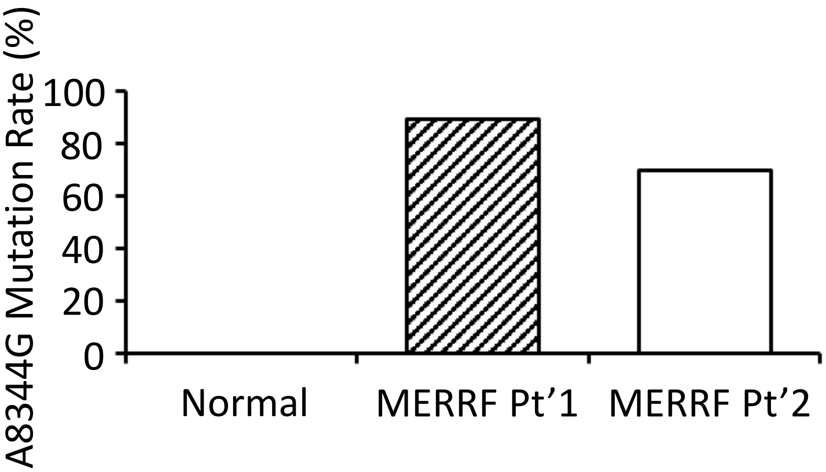

(B)

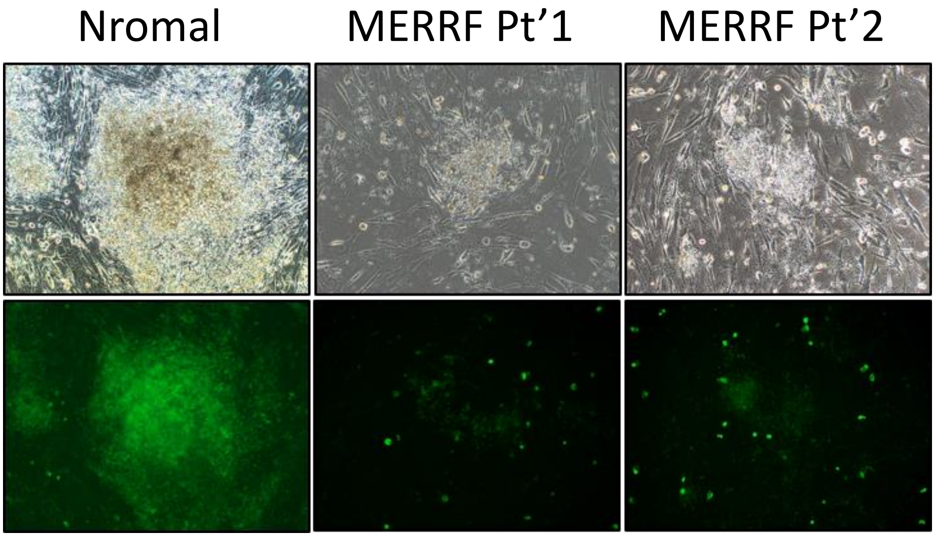

(C)

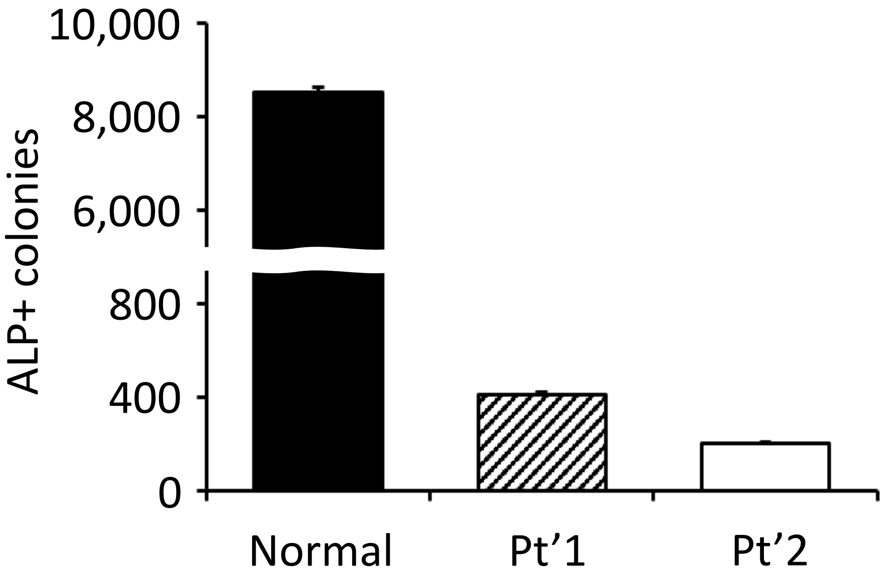

Supplementary Figure S1. mtDNA A8344G mutation downregulated the reprogramming efficiency. (A) mtDNA A8344G mutation rate was quantified by pyrosequencing. (B) Bright field and ALP positive colonies of generated hiPSCs. (C) Quantitative result of ALP positive colonies. Normal, healthy control. MERRF Pt'1, patient 1 with MERRF syndrome. MERRF Pt'2, patient 2 with MERRF syndrome.

Supplementary Table S1

Primer	Sequence	Tm ℃	Product Size	Accession Number
mtDNA A8344G Sequencing & Pyrosequencing				
mtF8203	5’-CATGCCCATCGTCCTAGAAT-3’	57.15	292 bp	NC_012920
mtR8394	Biotin-5’-TTTTATGGGCTTTGGTGAGG-3’	55.89		
Sequencing	5’-TAAGTTAAAGATTAAGAGA-3’	42.34		

Supplementary Table S2

Primer	Sequence	Tm ℃	Product Size	Accession Number
Stemness Marker				
endoOCT4-F	5’-CTTCAGGCACTGTGTTCATTG-3’	57.72	672 bp	NM_001159542
endoOCT4-R	5’-TTTGGCTGAACACCTTCCCA-3’	59.74		
endoSOX2-F	5’-GCCCTGCAGTACAACTCCAT-3’	60.04	735 bp	NM_003106
endoSOX2-R	5’-TTCCTGCAAAGCTCCTACCG-3’	60.04		
endoKLF4-F	5’-AGTTTCCCGACCAGAGAGA-3’	57.23	667 bp	NM_001314052
endoKLF4-R	5’-ACGCGAACGTGGAGAAAGAT-3’	60.04		
NANOG-F	5’-GAAGACAAGGTCCCGGTCAA-3’	59.60	709 bp	NM_024865
NANOG-R	5’-GGATTCAGCCAGTGTCCAGA-3’	59.38		
REX1-F	5’-GTGGGCCTTATGTGATGGCT-3’	60.11	759 bp	NM_174900
REX1-R	5’-TGCGTTAGGATGTGGGCTTT-3’	59.96		
DPPA2-F	5’-CCGTCCCCGCAATCTCCTTCCATC-3’	67.15	606 bp	NM_138815
DPPA2-R	5’-ATGATGCCAACATGGCTCCCGGTG-3’	67.64		
DPPA4-F	5’-TAGCACAGCAAAAGAGGCCA-3’	59.89	635 bp	NM_018189
DPPA4-R	5’-TGCATGGCCCATAAACAGGT-3’	59.96		
NAT1-F	5’-GGCTGCCCCAAGAGTGATAA-3’	59.74	550 bp	NM_001418
NAT1-R	5’-GTTGTTTGCTGCGGAGTTGT-3’	59.90		
Antioxidant Enzymes				
MnSOD-F	5’-GGCCTACGTGAACAACCTGAA-3’	60.54	71 bp	NM_000636
MnSOD-R	5’-CTGTAACATCTCCCTTGGCCA-3’	59.72		
CuZnSOD-F	5’-ACTTGGGCAATGTGACTGCT-3’	60.18	117 bp	NM_000454
CuZnSOD-R	5’-TTTCATGGACCACCAGTGTG-3’	58.02		
Catalase-F	5’-GCCTGGGACCCAATTATCTT-3’	57.24	118 bp	NM_001752
Catalase-R	5’-TAATTTGGAGCACCACCCTG-3’	57.79		
